# Transcultural adaptation and psychometric properties of Family Quality of Life Survey for caregivers of people with neurodegenerative disease: a study of Spanish families who live in the rural Spain–Portugal cross-border

**DOI:** 10.1186/s12955-021-01809-6

**Published:** 2021-06-30

**Authors:** Marta Badia, M. Begoña Orgaz, Isabel Vicario-Molina, Eva González-Ortega, María Gómez-Vela, Alba Aza, M. Antonia Martín-Delgado

**Affiliations:** 1 Institute on Community Integration (INICO), Faculty of Psychology, University of Salamanca, Avda. de la Merced, 109-131, 37005 Salamanca, Spain; 2 Teacher Training College of Zamora, University of Salamanca, Avda. Príncipe de Asturias s/n, 49029 Zamora, Spain; 3 Regional Health Management (RHM) of Castille and Leon (Spain), Paseo de Zorrilla, 1, 47007 Valladolid, Spain

**Keywords:** Family quality of life, Neurodegenerative diseases, *FQOLS-ND*, Cross-cultural adaptation, Psychometric properties

## Abstract

**Background:**

Neurodegenerative diseases (NDs) are one of the main causes of disability and dependence that have a great impact both on the quality of life of people with disabilities and their families. A majority of people with NDs receive care and support from the family, but there is no tool in Spain with which to measure whole-family QOL. The aim of this study was the translation, cultural adaptation, and validation of the *FQOLS–Dementia* into Spanish to assess FQOL among family members of individuals with NDs who live in the Spain–Portugal cross-border area.

**Method:**

The Spanish version was translated and adapted following the international guidelines for cross-cultural adaptation tests. A sample of 300 family caregivers was interviewed, applying an adapted version of the *Family Quality Survey* (*FQOLS–Dementia*). Confirmatory factor analysis was performed to validate the factor structure, and convergent validity was examined with Pearson’s correlation coefficients of the global FQOL with the domains. Internal consistency reliability was determined using Cronbach’s alpha.

**Results:**

The domain structure of the *FQOLS–ND* showed a good fit. In the convergent validity, it was found that the total score and the subscale domain scores were associated with the global FQOL score, except for the *Values* domain. Internal consistency of nine domain subscales was strong (α = 0.80 to 0.91), and excellent for the total FQOL (α = 0.85) and the global FQOL (α = 0.87).

**Conclusion:**

The *FQOLS–ND* presented good validity and reliability in caregiver families with individuals with ND, so its application shows its usefulness in detecting areas of improvement and intervention strategies for FQOL in the Spain–Portugal cross-border area.

**Supplementary Information:**

The online version contains supplementary material available at 10.1186/s12955-021-01809-6.

## Background

Neurodegenerative diseases (NDs) are one of the principal causes of disability and dependence worldwide, which have a major impact both on the health and psychological and social well-being of the people who suffer from them and their families [1]. The prevalence of NDs in Spain reaches 2.08% of the population, representing a total of 988,000 affected people [2]. Twenty-two percent of these people with NDs live in rural areas [2], regions characterized by a strong aging process and depopulation [3]. The prevalence of NDs in the rural cross-border Spain–Portugal population of the province of Salamanca (Castile and Leon, Spain) is 2.51%, slightly higher than in the Spanish population (2.08%) [4].

The caregiver family plays a crucial role in supporting the well-being of the person with ND, allowing them to live at home for as long as possible [3, 4]. Therefore, the family is one of the main providers of support services for the person with ND, often producing a high economic and social cost for the family unit [5]. In Spain, families assume most of the expenditure (87% of the total) of the needs of people with ND and dedicate an average of 70 h per week to caring for their relative [6].

Most of the studies have focused on the primary caregiver, finding burden care symptoms such as stress, anxiety, and depression [5–8]. However, little is known about how the person with ND affects the family as a whole [9–12].

In the 1990s, a paradigm shift took place, with the emergence of the family-centered model and intervention in people with developmental disabilities (DD) in the field of study [13–16]. At present, the Family Quality of Life (FQOL) paradigm has been consolidated and has become a reference for the organization and planning of services and programs for the families of people with DD [17–21].

The FQOL is a multidimensional social construct that reflects the positive values and life experiences of the family [15, 22, 23]. The FQOL is a dynamic sense of well-being of the family, collectively and subjectively defined and informed by its members, in which individual and family-level interact. That is, a family perceives quality of life (QOL) when its members’ needs are met, they enjoy their life together, and have opportunities to achieve goals that are transcendental for them [15]. More specifically, families experiment QOL when (a) they manage to carry out what they want, (b) they are satisfied with their attainment, and (c) they feel capable of living the life they want [24].

As in the field of study of family caregivers of people with DD, in the study and interventions in the field of NDs, a change in the approach is taking place, overcoming the classic conceptions based on the “model of deficits/stress”. In the new model, the “family quality of life model”, these families are considered to have coping difficulties, poor physical and social well-being, and feelings of guilt, and the objective of the model is to improve the quality of life of these families [11, 25, 26]. Caring for a person with ND not only leads to burden for the primary caregiver but also has adverse effects on family interactions and changes in family functions [10]. The family-systems approach emphasizes the interaction and interdependence among family members and emphasizes that any change, such as a health problem for one of the members, will have repercussions on all the others [27].

Recently, there has been considerable research on the conceptualization, measurement, and improvement of the quality of life of families, although much of the studies have focused on families of individuals with DD [28, 29]. The International Family Quality of Life Project was initiated in 1997 by researchers from Australia, Canada, and Israel. It examines the quality of life of families who have one or more members with a DD to advance in the approach to the FQOL construct and develop an evaluation tool [13]. Currently, this project involves the collaboration of a team of researchers from several countries around the world. For these experts, the principles that guide the application of the conceptual model of FQOL are: (1) FQOL is a multidimensional construct and influenced by several factors; (2) it comprises the same dimensions for all people; (3) it includes objective and subjective components; and (4) it is best studied using qualitative and quantitative methodology [13]. Five factors were identified as contributing to FQOL in families providing care to people with dementia: (1) family interactions, (2) support of direct care/activities of daily living; (3) emotional/behavioral well-being; (4) physical and cognitive well-being; and (5) disability support/medical care [25].

To assess the degree to which FQOL is enjoyable, meaningful, and supported by resources that are important to all family members, the FQOL project developed the *Family Quality of Life Survey* (*FQOLS–2006*) [30–32]. This tool collects quantitative data (on Likert scales) and qualitative data (through open questions) on six measurement dimensions (*Importance*, *Opportunities*, *Initiative*, *Stability*, *Attainment*, and *Satisfaction*) in nine domains of FQOL (*Health of the family*, *Financial well-being*, *Family relationships*, *Support from other people*, *Support from disability-related services*, *Influence of values*, *Careers, Leisure and recreation*, and *Community interaction*). The final section of the survey includes items about overall impressions of FQOL [30]. The survey had good reliability and validity for caregivers of people with DD [31–33]. Using data from Australia, Canada, and the United States, it was reported that the four indicators (*Initiative*, *Opportunities*, *Attainment*, and *Satisfaction),* and the nine-domain factor structure of the FQOLS-2006 had an acceptable level of construct validity [*χ*^2^ (27) = 55.32, *p* < 0.00, CFI = 0.93, AGFI = 0.94 RMSEA = 0.06] [31]. Concurrent validity showed moderate correlations between five FQOLS-2006 domains and conceptually related domains. Cronbach's alpha internal consistency of the nine domains ranged from poor to good (0.43 to 0.83) and, for the two-item global FQOL, it was 0.85 [33].

The *FQOLS–2006* was designed to evaluate FQOL in families that have a member with DD, but recently, there is a new version for primary caregivers of people with dementia [34]. Modifications of the original survey included: (a) the adaptation of language to reflect the perspective of a caregiver of a person with dementia; (b) the incorporation in the *About your family* section of thirteen items and five categories of responses that measured the level of independence in six activities of daily life (i.e., eating, grooming, dressing, etc.) and five practical activities (i.e., housekeeping, shopping, money management, etc.); and (c) the differentiation between practical support and emotional support in the area *Support from other people* [34]. The internal consistency was high for two item-item global FQOL scale (Cronbach's alpha = 0.85) and for the *FQOLS–Dementia* domain scales, it ranged from poor to good (Cronbach's alpha range = 0.56–0.85). The domain-level outcomes of the scales’ internal consistency were considered good [9].

This study aimed to: (1) translate and culturally adapt the *FQOLS–Dementia* into Spanish, to assess the FQOL among family members of individuals with NDs who live in the cross-border area Spain–Portugal; and (2) further examine the specific psychometric properties of reliability and validity.

## Method

### Participants and study setting

Participants were recruited from Regional Health Management (RHM) of Castille and Leon (Spain), between October 2019 and July 2020. Family members of patients with NDs were invited to participate in the study if they met the following inclusion criteria: (1) being a family member of a person with an ND; (2) contributing to the daily care of the person with ND but not necessarily the primary caregiver; (3) being 18 years of age or older; (4) providing their consent; and (5) residing in the cross-border area of Spain–Portugal (Salamanca, Castille Leon). Families whose relative with an ND lived in residential accommodation were excluded.

**Fig. 1:**
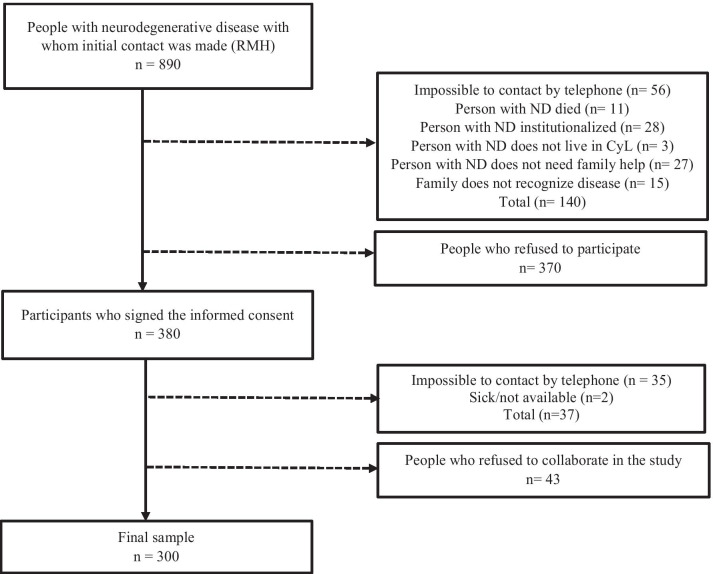
Sample recruitment process

The sample size (*n* = 348) was calculated through the prevalence of NDs in the cross-border area Spain–Portugal in 2020 (*N* = 987; Dementia: 58.7%, Parkinson: 37.37%, Multiple Sclerosis: 3.7%), using statistic tables [35]. The RHM selected 890 families of patients with ND, and 380 families signed the informed consent to participate. The final sample was composed of 300 participants (Fig. 1).

### Instrument: The Family Quality of Life Survey–Dementia

The *FQOLS–Dementia* instrument was developed to measure the QOL of families with at least one member with dementia [34]. This survey has several parts. The first one, *About your Family,* collects descriptive information about the family. The next nine parts ask about specific domains of family life, concretely, *Family Health*, *Financial well-being, Family relationships*, *Support from others*, *Support from services*, *Values*, *Careers*, *Leisure and recreation*, and *Community interaction*. Each part contains two sections. Section A addresses the context and general information, and Section B assesses the *importance*, *opportunities*, *initiative*, *stability, attainment*, and *satisfaction* with the specific domain of family life. While *attainment* and *satisfaction* are considered outcome dimensions, *importance, opportunities, initiative,* and *stability* are explanatory dimensions [13]. All Section B items are rated on a 5-point Likert scale ranging from 1 (*Hardly important at all*) to 5 (*Very important*). The last part of the instrument asks about general impressions related to FQOL. The reliability of the *attainment* and *satisfaction* dimensions of the *FQOLS–Dementia* ranged from moderate (0.56) to good (0.85) [9].

### Translation and adaptation process

The process of translation, adaptation, and validation of the *FQOLS–Dementia* [9] to the Spanish context for families caring for people with an ND (*FQOLS–ND*) was carried out following the guidelines and recommendations for test adaptation proposed by the International Testing Commission (ITC) [36, 37].

The translation and adaptation process was performed in 6 phases: (1) translation of each item to Spanish by two different translators with knowledge and experience in the field of FQOL. For each item, the translators indicated the degree of difficulty and the level of equivalence of the translation on a scale ranging from 1 to 10; (2) synthesis of the translations by agreement of the two translators, resulting in the first Spanish version of the original instrument; (3) selection of two focus groups, one with four families of an individual with an ND and another group with eight professionals of social services to analyze the cultural acceptability, make suggestions, and ensure the suitability of the items; (5) concordance and synthesis was performed by a committee of experts made up of four researchers of NEUROQUALYFAM team (cross-border Spain–Portugal cooperation project with the support of the European Union–POCTEP) to achieve the semantic, idiomatic, and conceptual equivalence of the survey; (6) the backward translation of the adapted instrument was carried out by a PhD in Psychology, whose maternal language is English and who is bilingual in Spanish to compare the differences or discrepancies with the original version, thereby obtaining the definite version of the *FQOLS–ND*.

### Data collection and procedure

The identification of potential participants was carried out by the Regional Management of Health (RMH) of Castille and Leon, which sent certified letters to the selected families with information about the study and the request for them to consent to participate, and to the coordinator of the Primary Care Management of Salamanca province to present the research to the families of patients with ND.

As a consequence of the COVID-19 pandemic, data collection was redesigned because the length of the tool made it difficult to administer. For this reason, only the *attainment* (the degree to which the family can obtain the things that it wants) and *satisfaction* (the overall perception of important aspects of family life) dimensions were evaluated. The decision to use these two dimensions was based on the following three considerations: (1) from a psychometric perspective, these dimensions obtained excellent internal consistency (Cronbach's alpha = 0.85) in the validation study of the *FQOLS–2006* [31]; (2) from a conceptual perspective, *attainment* and *satisfaction* are considered as outcome indicators of FQOL [31]; and (3) when validating the *FQOLS–Dementia,* only *attainment* and *satisfaction* were examined, obtaining Cronbach's alpha coefficients comparable to the original *FQOLS–2006* [9].

To respect the social distancing restrictions, the questionnaires were administered via telephone by trained and experienced interviewers. Verbal informed consent was obtained after informing the participants about the aim of the study and their right to drop out at any time. Completing the survey took approximately 30 min.

### Statistical analysis

Data were analyzed using SPSS 26.0. First, descriptive statistics of the participants’ characteristics were calculated. The *feasibility of the survey* was determined by the response rate, the time spent to complete the questionnaire, the percentage of missing values per item, the distribution of scores, and the frequency of the maximum and minimum values registered.

A descriptive analysis of the items was conducted. Distribution of scores for each item, proportion of missing data, the proportion of “does not apply” responses, and floor and ceiling effects were examined. Floor and ceiling effects were considered to occur when more than 15% of the respondents endorsed the lowest (1) or highest (5) scores, respectively [38].

#### Data computation

Domain subscale total scores were computed as the mean of each domain’s two-dimensional ratings: *attainment* and *satisfaction*. In the case of the *Others* domain, the mean of the two dimensions, Emotional and Practical Support, was calculated. This version of the survey generates two types of FQOL scores: (a) the total score, computed by aggregating the 20 items of Section B (i.e., two dimensions of each of the eight domains, and four items from the *Others* domain); and (b) the global score, computed from the mean of the two items related to the overall FQOL.

#### Differences between domains and between dimensions

A descriptive analysis of the domains and dimensions was conducted. A two-factor (domains and dimensions) repeated-measures ANOVA was used, and post hoc comparisons were used to compare the domains and analyze the interactions. The significance level was set at 0.05. Concerning the interpretation of the effect size (η_p_^2^), we used guidelines proposed by Cohen (0.01 = small effect, 0.06 = moderate effect, 0.14 = large effect) [39].

#### Construct validity

We determined whether FQOL is a latent variable that can be measured with the nine domains as indicators [13, 31], using Confirmatory Factor Analysis (CFA), with the statistical package AMOS v.18. Maximum Likelihood (ML) techniques were used to estimate model parameters, similar to analyses undertaken by other authors [31, 40], and the model’s goodness-of-fit and the significance of the model’s parameter estimates were calculated.

Model fit was evaluated using a chi-square test, the Root Mean Squared Error of Approximation (RMSEA < 0.06) and the Comparative Fit Index (CFI > 0.95) [41]. Standardized factor loadings (λ), squared multiple correlations (SMC), and modification indices were examined to determine whether the indicators contributed significantly to the model. Indicators with low factor loadings (λ < 0.50) were considered to make a low contribution to the model.

#### Convergent validity

Pearson’s correlation coefficient values of the domains scores and the total score with the Global FQOLS–ND score were evaluated to examine convergence. Correlation coefficients of about 0.10 were considered to be small, 0.30 medium, and 0.50 large [39].

#### Reliability assessment

To evaluate internal consistency, Cronbach’s alpha coefficient was calculated for the nine domains. An alpha value between 0.70 and 0.95 was considered satisfactory [42].

## Results

### Translation and cultural adaptation

The survey included some minor modifications. First, a change of term (dementia is replaced by neurodegenerative disease) and modifications related to the wording of the questions to reflect the perspective of families caring for people with ND. Second, this work is part of the NEUROQUALYFAM project, which studies QOL among families with a member who has an ND in the Spain–Portugal cross-border area. For this purpose, we removed the open question items due to the difficulty to code them, whereas the shorter extension of the instrument facilitated its application. Third, in the part *About Your Family*, one item was added to assess the person’s officially recognized degree of dependency to carry out basic daily life activities. Fourth, in Section A, minor adjustments were made. For example, in Item 1 of the *Services* dimension, the different response options to the available support resources for the person with ND were adapted to the Spanish context. Lastly, in Section B, all 60 items of the *FQOLS–ND* are the same as in the original version. The only change was to replace the term dementia with neurodegenerative disease.

The final version of the *FQOLS–ND* contains the following parts. The first part, *About your family*, includes 12 questions about the family and the person with ND. The questions range from general socio-demographic issues to more specific ones, such as the supports needed by the relative with ND or the degree of independence in different daily life activities. The second part contains the same nine domains of the FQOL of the original survey: *Family health, Financial well-being*, *Family relationships*, *Support from others*, *Support from services*, *influence of Values*, *Careers*, *Leisure and recreation*, and *Community interaction*. Each of these nine parts has 2 sections: Section A is composed of 33 quantitative items about specific issues within each of the nine core domains of the FQOL. Section B, in each of the nine life domains, six dimensions (or indicators) are used to examine how the family perceives its FQOL. These dimensions include *importance*, *opportunities*, *initiative*, *stability*, *attainment*, and *satisfaction*. This section consists of 60 items and collects quantitative data of each of the nine life domains on a 5-point Likert scale, with higher scores indicating higher levels of the specific domain. The final section *Overall Family Quality of Life* consists of two closed-ended questions about global impressions of the FQOL (Additional file 1).

### Descriptive characteristics of the study sample

Three hundred participants completed the study. Their characteristics are shown in Table 1. The mean age is 62.4 years (*SD* = 13.34, range = 25–88 years), with almost 60% aged 65 or above. The majority are females (70%), married/living with a partner (79.7%), unemployed (64.7%), with low income –up to 1000 EUR per month– (66.1%), and have elementary or high school qualifications (72.8%). The vast majority are either the spouse/partner (40.9%) or son/daughter (51.7%) of the care-recipient, and are the primary caregiver (93.3%), with three out of four living in the same household as the care-recipient. Most of them live in rural areas of up to 500 (44%) or 500–10,000 people (35.7%).

**Table 1:** Family caregiver characteristics (n = 300)

Variable	n	%
Age (M = 62.48, DT = 13.34, Range = 25–88)		
Up to 65 years	178	59.3
More than 65 years	122	40.7
Gender		
Male	90	30.0
Female	210	70.0
Educational level		
No school certificate	21	7.0
Elementary school	150	50.0
High school	68	22.8
University	59	19.8
Employment status		
Working (employees + self-employed)	106	35.3
Not working (retired + unemployed + others)	194	64.7
Income (EUR per month)		
Up to 500	95	31.9
500–1000	102	34.2
1000–1500	69	23.1
More than 1500	32	10.7
Marital status		
Married or with partner	239	79.7
Others (divorced or separed. widowed. single)	61	20.3
Place of residence—number of in habitants		
More than 10.000	61	20.3
500–10.000	107	35.7
Up to 500	132	44.0
Relationship with person with dementia		
Spouse or partner	117	40.9
Son/Daughter	148	51.7
Others	21	7.3
Primary caregiver		
Yes	280	93.3
No	20	6.7
Living condition		
Living with patient	225	75.0
Not living with patient	75	25.0

Concerning the characteristics of care-recipients (Table 2), their mean age is 79.3 years (*SD* = 11.7, range = 20–98), and most are females (60%). All of them suffer from dementia (54.3%), Parkinson´s disease (26.7%), or multiple sclerosis (6.7%) with some degree of dependence [43] (67.3%)—generally high (46.2%) or moderate (30.7%). Concerning support, 91% have support needs, and in 55.4%, these needs are high. Of these people, 48% cannot maintain a coherent conversation.

**Table 2:** Care-recipient characteristics (n = 300)

Variable	n	%
Age (M = 79.3, DT = 11.7, Range = 20–98)		
Gender		
Male	120	40.0
Female	180	60.0
Diagnosis		
Dementia	163	54.3
Parkinson Disease	80	26.7
Multiple Sclerosis	20	6.7
Others (unknown by family; several NDs)	37	12.3
Dependence		
Yes	202	67.3
No	98	32.7
Grade of dependence		
Grade 1	46	23.1
Grade 2	61	30.7
Grade 3	92	46.2
Supports needed		
None	27	9.0
Very few	32	10.7
Some	75	25.0
Quite a lot	56	18.7
A lot	110	36.7
Communication skills		
Poor communication	76	25.3
Only basic needs	26	8.7
Needs, desires, ideas	42	14.0
Coherent on some topic	66	22.0
Cooherent on many topics	90	30.0

### Descriptive characteristics of the FQOLS–ND

The percentage of missing data for each item was 0%. The minimum and maximum scores in every item were 1 and 5 (only in the dimensions of *attainment* and *satisfaction* of the domain Family Health had a minimum score of 2).

A ceiling effect in the domains of *Family* (53.0%) and *Careers* (19.0%) was observed for the *attainment* dimension. A ceiling effect was found for the *satisfaction* dimension in the domains of *Family* (50.7%), *Others* (18.7%), and *Careers* (20.0%).

Skewness ranged from − 1.13 to − 0.17 and from − 1.40 to − 0.23 for *attainment* and *satisfaction*, respectively. Kurtosis ranged from − 0.13 to 1.38 (*attainment*) and from − 0.61 to 2.56 (*satisfaction*).

The total *FQOLS–ND* scores were virtually symmetrical (− 0.45 and − 0.85, for *attainment* and *satisfaction*, respectively), and slightly leptokurtic (1.39 and 1.38, for *attainment* and *satisfaction*, respectively) (Table 3).

**Table 3:** Descriptive characteristics of the *FQOLS–ND*

Domains	Attainment					Satisfaction				
	*M* (*SD*)	Skewness	Kurtosis	% Floor	% Ceiling	*M* (*SD*)	Skewness	Kurtosis	% Floor	% Ceiling
Health	3.64 (0.69)	− 0.52	0.20	0.0%	6.0%	3.66 (0.84)	− 0.72	0.42	1.0%	11.0%
Finances	3.53 (0.64)	− 0.86	0.93	0.7%	1.3%	3.58 (0.69)	− 1.19	1.46	1.0%	2.0%
Family	4.41 (0.72)	− 1.13	1.38	0.3%	53.0%	4.35 (0.80)	− 1.40	2.00	0.7%	50.7%
Others	3.19 (1.14)	− 0.53	− 0.50	12.3%	8.3%	3.76 (0.87)	− 0.44	0.03	1.0%	18.7%
Services	2.83 (1.02)	− 0.29	− 0.61	13.7%	2.3%	3.11 (1.11)	− 0.56	− 0.61	12.7%	5.0%
Values	3.49 (0.86)	− 0.17	0.53	2.7%	13.0%	3.64 (0.82)	− 0.23	0.46	1.7%	15.0%
Careers	3.96 (0.71)	− 0.71	1.30	0.3%	19.0%	3.98 (0.75)	− 1.12	2.56	1.0%	20.0%
Leisure	3.25 (0.98)	− 0.66	− 0.13	6.7%	4.3%	3.43 (0.95)	− 0.92	0.42	5.3%	6.0%
Community	3.74 (0.80)	− 0.93	1.29	1.3%	11.7%	3.83 (0.71)	− 0.89	1.80	0.7%	11.7%
Total scores	3.52 (0.49)	− 0.45	1.39			3.71 (0.49)	− 0.85	1.38		

Health, Health of family; Finances, Financial wellbeing; Family, Family relationships; Others, Support from other people; Services, Support from disability-related services; Values, Influence of values; Careers, Careers and preparing for careers; Leisure, Leisure and recreation; Community, Community interaction

*M* Mean,* SD* Standard deviation

### Differences between domains and between dimensions

The means in *attainment* and *satisfaction* in each of the nine domains are shown in Table 3. *Attainment* and *satisfaction* mean ratings were similar across the nine domains.

The ANOVA revealed significant differences between the domains, *F*(8, 2392) = 96.77, *p* < 0.001, η_P_^2^ = 0.25. The domains that participants perceived as significantly higher (*p* < 0.001) were *Family* (*M* = 4.38, *SD* = 0.71) and *Careers* (*M* = 3.97, *SD* = 0.69), whereas the domains perceived as significantly lower (*p* < 0.001) were *Services* (*M* = 2.97, *SD* = 0.99) and *Leisure* (*M* = 3.34, *SD* = 0.92).

When comparing the domains separately in the dimensions of *attainment* and *satisfaction*, results revealed significant differences between scores for the domains in *attainment, F*(8, 2392) = 108.45, *p* < 0.001, η_P_^2^ = 0.27, and *satisfaction, F*(8, 2392) = 64.98, *p* < 0.001, η_P_^2^ = 0.18. The domains that participants perceived as significantly higher (*p* < 0.001) in *attainment* were *Family* (*M* = 4.41, *SD* = 0.72) and *Careers* (*M* = 3.96, *SD* = 0.71), whereas the domains perceived as significantly lower (*p* < 0.001) in *attainment* were *Services* (*M* = 2.83, *SD* = 1.02) and *Others* (*M* = 3.19, *SD* = 1.14). In terms of *satisfaction* with the domains, the participants reported significantly higher *satisfaction* (*p* < 0.001) in the domains of *Family* (*M* = 4.35, *SD* = 0.80) and *Careers* (*M* = 3.98, *SD* = 0.75), whereas *Services* (*M* = 3.11, *SD* = 1.11) and *Leisure* (*M* = 3.43, *SD* = 0.95) were perceived as significantly less satisfactory (*p* < 0.001).

Results indicated a statistically significant difference between the dimensions, *F*(1, 299) = 118.96, *p* < 0.001, η_P_^2^ = 0.29. The mean level of *satisfaction* experienced (*M* = 3.71, *SD* = 0.49) was significantly higher than mean level of *attainment* (*M* = 3.52, *SD* = 0.49).

The interaction Domains x Dimensions was significant, *F*(8, 2392) = 32.69, *p* < 0.001, η_P_^2^ = 0.10, and the a posteriori tests revealed that the mean level of *satisfaction* was higher than the mean ratings of *attainment* in eight of the nine domains, although these differences were only statistically significant in six domains: *Finances* (*p* = 0.03), *Others* (*p* < 0.001), *Services* (*p* < 0.001), *Values* (*p* < 0.001)*, Leisure* (*p* < 0.001), and *Community* (*p* = 0.001). The participants reported a higher level of *attainmen*t only in the *Family* domain (*p* = 0.04).

Large effect sizes were found in the domain and dimension factors. However, the effect size of the interaction was medium. A large effect size was also obtained when analyzing the differences between domains separately in the dimensions of *attainment* and *satisfaction*.

### Construct validity of FQOLS–ND

#### Factor structure

Results showed that the hypothesized model, consisting of nine indicators (the nine domain subscales) and one latent factor presented a moderate fit, χ^2^(27) = 99.21, *p* < 0.001, CFI = 0.84, RMSEA = 0.10.

These results reflect the cultural values of the society in our study, in which the families and close relatives of the person with an ND are characterized by commitment, solidarity, and the family obligation to provide care to the dependent relative [44]. The results also show that the very high economic costs of dementia in Spain are borne by the family, and highlight the impossibility or difficulties to access social-health resources because they are either insufficient or economically inaccessible [44]. For this purpose, an inter-correlation between indicators was proposed, which means that these indicators are related to each other because they all share the quality of representing FQOL. More specifically, correlations were proposed between *Others* and *Values, Family* and *Values,* and *Health* and *Finances*.

The inclusion of the error covariance between the domains of *Health* and *Finances*, *Values,* and *Family*, and of *Values* and *Others* improved model fit, χ^2^(24) = 31.48, *p* = 0.14, CFI = 0.984, RMSEA = 0.032.

**Fig. 2:**
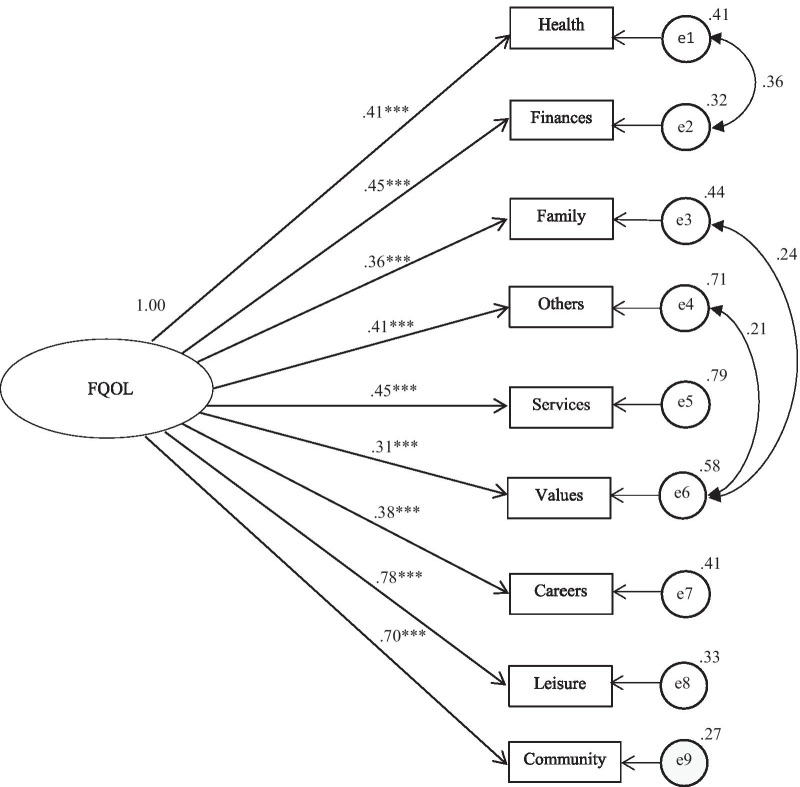
Domain structure of total *FQOLS–ND*

All the indicators were statistically significant (*p* < 0.001), as shown in Fig. 2, *Leisure* (λ = 0.78) and *Community* (λ = 0.70) had the highest factor loadings, whereas *Values* (λ = 0.31) *Family* (λ = 0.36), and *Careers* (λ = 0.38) had the lowest factor loadings. *Leisure* (61%) and *Community* (49%) also showed the highest contribution to the variance of the latent factor of the FQOL, whereas the contribution of the remaining domains was lower than 30%: *Services* and *Financial* (20%), *Health* and *Others* (17%), *Careers* (14%), *Family* (13%) and *Values* (9%).

#### Correlations between the domain subscales

Correlations between the domain subscales ranged from weak (*r* = 0.06, *p* > 0.05, the correlation between *Finances* and *Values,* which did not reach statistical significance) to strong (*r* = 0.55, *p* < 0.001, the correlation between *Leisure* and *Community*). See Table 4.

**Table 4:** Correlation matrix and Cronbachs’s α of FQOL domains

	1	2	3	4	5	6	7	8	9	10
1. Health	**.82**									
2. Finances	.48***	**.89**								
3. Family	.23***	.18**	**.85**							
4. Others	.12*	.15**	.16**	**.80**						
5. Services	.14*	.26***	.12*	.23***	**,85**					
6. Values	.13*	.06	.33***	.31***	.13*	**.90**				
7. Careers	.20***	.14*	.23***	.20***	.08	.24***	**.88**			
8. Leisure	.35***	.36***	.26***	.27***	.36***	.23***	.29***	**.90**		
9. Community	.26***	.29***	.24***	.33***	.31**	.21***	.23***	.55***	**.91**	
10. Global FQOL	.42***	.39***	.25***	.21***	.26***	**.11**	.31***	.51***	.43***	**.87**
Mean (SD)	3.65 (0.71)	3.56 (0.63)	4.38 (0.71)	3.48 (0.93)	2.97 (0.99)	3.57 (0.80)	3.97 (0.69)	3.34 (0.92)	3.79 (0.71)	3.67 (0.68)

Cronbach’s α of the FQOL domain subscales are presented on the diagonal

^*^p < .05; **p < .01; *p* < .001

### Convergent validity

Total and subscale (domain) scores were significantly correlated (*p* < 0.001) with the Global FQOL score, except for the *Values* domain. The correlation coefficient for the total and Global FQOL scores was large (*r* = 0.55). The magnitude of the correlation coefficients of the Global FQOL score and the domains, in descending order, were: large for *Leisure* (*r* = 0.51); medium for *Community* (*r* = 0.43), *Health* (*r* = 0.42), *Finances* (*r* = 0.39), and *Careers* (*r* = 0.31); and small for *Services* (*r* = 0.26), *Family* (*r* = 0.25), and *Others* (*r* = 0.21) (Table 4).

### Reliability

As shown in Table 4, the internal consistency of the nine domain subscales, containing two items each, was satisfactory, with alphas ranging from Cronbach α = 0.80 to 0.91. Moreover, internal consistency was excellent for the total 18-item FQOL scale (Cronbach's alpha = 0.85) and for the Global FQOL scale, containing two items related to the overall FQOL (Cronbach's alpha = 0.87).

## Discussion

The purpose of this study was to translate, culturally adapt, and validate the *FQOLS–Dementia* that takes into account the characteristics of the target population, families who care for people with NDs in the cross-border area Spain–Portugal.

The analysis of the scores found almost no ceiling or floor effects in the dimensions within each of the domains, with 15% considered the maximum acceptable. However, on the one hand, the *Family* and *Careers* domains had a ceiling effect in the *attainment* dimension. This indicates that the family perceives that can it achieve quality relationships among the different family members. That is, the family achieves a high degree of support, respect, and mutual trust, and can reach agreements and decisions, and remains together. This result is similar to that obtained by previous studies that consider that affection and solidarity among family members in the care of dependents are aspects of great value [11, 19]. Moreover, unlike in previous studies, caring functions performed by the families do not imply giving up the academic and professional career of any of its members [2, 45]. On the other hand, the *satisfaction* dimension had a ceiling effect in the *Family, Others,* and *Careers* domains, which reflects the family’s high perception of family cohesion, support from the close environment, and opportunities for vocational training and professional qualification. These results are similar to those reported by other authors who point out that the family system is a platform of essential resources to deal with the caring tasks of a family member with dementia, and family caregivers who have a greater social support network reported a better QOL [44, 46].

Likewise, the present study examined two outcome measures for each of the nine family life domains of the *FQOLS–ND*. The *attainment* and *satisfaction* means were similar in all nine domains, albeit the *Family* and *Careers* domains were rated higher, and the *Services* and *Leisure* domains were rated lower. These results have been partially confirmed by previous studies that indicate, on the one hand, the positive effect of quality family relationships and, on the other hand, the low effect that caring for the person with an ND has on the family’s training and work activities. They also show the difficulties that the family has to access adequate professional services and enjoy leisure activities [9, 18]. Also, the results have shown that the *satisfaction* scores were higher than the scores in *attainment,* and they were significant in *Finances, Others, Services, Values, Leisure*, and *Community*. As is well known, satisfaction scores are often over-estimated because family caregivers tend to report moderately positive levels of well-being, except in rare cases where they feel overwhelmed to the point of not being able to cope with daily challenges [47]. Finally, a noteworthy result was that families reported a higher level of attainment than satisfaction in the *Family* domain. This may indicate that, despite having achieved good relationships among family members and high levels of co-responsibility in decision-making and problem-solving concerning their caring functions, this was at the cost of a reduction in well-being. In short, the caring family, beyond direct physical care (e.g., administering medication, assisting in daily grooming, preparing meals, etc.), must organize and distribute care responsibilities (e.g., managing health services, care planning, accompaniment to doctors, etc.), which can have negative consequences on family satisfaction [44, 45].

The CFA provides empirical support for the construct validity of the *FQOLS–ND* for the population of caring families in the cross-border Spain–Portugal area. The CFA allowed us to establish the validity of this scale and supports the FQOL construct proposed by the authors of the original version of the instrument [31]. The CFA produced fit indices clearly suitable for the nine-domain model, better than those obtained by previous studies [31, 40]. We also found a high contribution of the *Leisure* and *Community* domains to FQOL, a similar result to that obtained by a previous study on the psychometric properties of the *FQOLS–2006* [40]. That is, the family’s opportunities to participate and enjoy leisure activities and free time, as well as their involvement in social life, are factors that lead to better results of FQOL, results that are confirmed by previous scientific literature [46, 48–52]. In short, these results allow us to conclude that the structure of a latent factor, FQOL, represented by the nine domains, is replicated.

In this study, we present the domain structure of the total FQOL as a latent variable that can be measured using nine indicators for each domain [40]. We agree with the authors of the scale that the value of this scale is that it gathers information about a large number of domains or areas of the family in which the family's needs and support resources can be detected, although some domains are less closely related to the other domains or to the total or global scores. Also, the tool is not intended to reduce the FQOL to a single score, but rather to provide information about each of the nine domains and to facilitate the identification of needs at the individual family level, to contribute to the improvement of the services and supports the families receive [40].

The convergent validity, tested by the relationships between the domain scores and the global FQOL score, showed a strong association of this score with *Leisure* and a moderate one with the domains of *Community* and *Health,* like the results obtained in prior research on family caregivers of people with dementia [9]. In the face of care demands required by a person with ND, caregiver families often restrict their participation in enjoyable activities and have few opportunities to maintain social networks, which can affect family well-being [9, 51]. *Values* was the only domain that was not directly associated with the global FQOL score. In summary, the positive correlations between the different subscales and the global FQOL score may mean that, in FQOL, satisfaction is measured similarly by the perceptions of the eight domains.

Internal consistency of the *FQOLS–ND* subscales was found to be excellent. Internal consistency of the 20-item total FQOL scale was also excellent. Internal consistency results are similar to those found in other studies using the original version of the instrument in caring families of people with DD and dementia [9, 31]. However, it should be noted that the internal consistency rates of this study are somewhat higher and especially more significant in the *Health, Finances,* and *Services* subscales*.* The higher Cronbach alpha values obtained in our study, despite including a lower number of ítems, are explained because all the items included in our scale correspond to the outcome dimension (*attainment* and *satisfaction*).

The practical implications of the results obtained concerning the levels of evaluation, intervention, and planning of support services are multiple. Thus, the *FQOLS–ND* allows us to evaluate the FQOL profile of caring families of people with ND, identifying their needs and priorities. More specifically, it provides an assessment of the integral needs of the caring family, which serve as a guide for the design of comprehensive intervention plans, offering the necessary supports for the family to perform their caring functions with less discomfort and better FQOL. Finally, the *FQOLS–ND* is a useful tool for planning, organization, and evaluation of quality social healthcare services for people with ND and their families living in rural areas of the cross-border Spain–Portugal area.

The Coronavirus disease (COVID-19) affected the data collection for this study. One of the challenges was to review the contents of the instrument to develop a shorter version of the scale and facilitate the collection of the respondents' data. The translated and adapted Spanish version of the *FQOLS–Dementia* for a population with ND is an overly long instrument, as the authors of the original instrument point out [9]. For this reason, as indicated in the data collection and procedure sections, we chose to include only the *attainment* and *satisfaction* dimensions for the nine domains of FQOL to analyze the properties of the instrument.

Therefore, this study has some limitations. We could not analyze the factorial structure at two levels, like the authors of the original scale. Specifically, we could not analyze the item-level factor structure of the domains, the domain subscale aggregated from six dimensions, because only items corresponding to the dimensions of *attainment* and *satisfaction* were included in this study. On the other hand, the model we present is a model of FQOL outcome measures because the explanatory measures (*importance*, *opportunities*, *initiative*, and *stability*) were not included.

Finally, beyond the psychometric properties, a scale and user manual will be developed for the Spanish version to illustrate the scores in an FQOL profile that will facilitate the interpretation of the scores.

## Conclusion

This study has provided evidence of the validity and reliability of the *FQOLS–ND* to assess the QOL of caring families of people with an ND in the cross-border Spain–Portugal area. The findings highlight the importance of family involvement in leisure activities and community integration to increase their QOL. This tool's usefulness for improving FQOL results is noteworthy, implementing evidence-based practices and guiding the planning of support services.

## Supplementary Information

**Additional file 1**. Escala de Calidad de Vida Familiar: Enfermedades neurodegenerativas.

## Data Availability

Data sharing is available upon reasonable request. Kindly contact the corresponding author.

## Abbreviations

RHM: Regional Management of Health
CyL: Castille and Leon
CFA: Confirmatory factor analysis
ML: Maximum Likelihood
CFI: Comparative Fit Index
AGFI: Adjusted Goodness-of-Fit Index
RMSEA: Root Mean Square Error of Approximation
SMC: Squared Multiple Correlations
λ: Standardized factor loadings
