# Supplementary material for: Transcultural adaptation and psychometric properties of Family Quality of Life Survey for caregivers of people with neurodegenerative disease: a study of Spanish families who live in the rural Spain–Portugal cross-border
Source: Health Qual Life Outcomes. 2021 Jun 30;19:172. doi: 10.1186/s12955-021-01809-6 (PMC8242286; doi:10.1186/s12955-021-01809-6)
Supplement: Supplementary file 1 — Additional file 1. Escala de Calidad de Vida Familiar: Enfermedades neurodegenerativas. [file 12955_2021_1809_MOESM1_ESM.pdf]

Escala de Calidad de Vida Familiar

Personas con enfermedades neurodegenerativas

2021

Esta versión de la escala fue traducida, modificada y adaptada de la Family Quality of Life Survey Family Quality of Life Survey-Dementia © 2011. Rosanne DiZazzo-Miller y Preethy S. Samuel.

Marta Badia
M.ª Begoña Orgaz
Isabel Vicario-Molina
Eva González-Ortega
María Gómez-Vela
Alba Aza
NEUROQUALYFAM Group

Coordinated by:

Project "NEUROQUALYFAM, Neurodegenerative Diseases and Familay Quality of Life", funded by the Fondo Europeo de Desarrollo Regional (FEDER) through the program INTERREG V-A España – Portugal (POCTEP) 2014 – 2020.

Escala de Calidad de Vida Familiar: Enfermedades neurodegenerativas

Marta Badia, M.ª Begoña Orgaz, Isabel Vicario-Molina, Eva González-Ortega, María Gómez-Vela, Alba Aza y NEUROQUALYFAM Group.

Primera impresión abril 2021

Reproducción y Condiciones de Uso:

La *Escala de calidad de vida familiar: Enfermedades neurodegenerativas* está pensada para su uso internacional y puede imprimirse o copiarse con fines educativos, de servicios o de investigación sin permiso de los autores. De cualquier modo, los autores aprecian y animan vivamente a los educadores e investigadores a informarles de los usos que se den a la *Escala de calidad de vida familiar: Enfermedades neurodegenerativas* y les invitamos a compartir los datos y análisis. Cualquier otro uso o adaptaciones de la escala requieren permiso escrito de Barry J. Isaacs en la dirección indicada abajo.

Correspondencia e información

Para consultas generales:	Para compartir datos	Para su aplicación en España:
Surrey Place Centre<br>2 Surrey Place<br>Toronto, Ontario<br>M5S 2C2<br>barry.isaacs@surreyplace.on.ca	Rosanne DiZazzo-Miller<br>Assistant Professor<br>Wayne State University<br>Occupational Therapy Program<br>259 Mack Ave, Suite 2212<br>Detroit, MI 48202<br>Ar7975@wayne.edu	Marta Badia<br>Eva González-Ortega<br>INICO<br>Facultad de Psicología<br>Avda. de la Merced 109-131<br>37005 Salamanca<br>España<br>badia@usal.es<br>evagonz@usal.es

Escala de Calidad de Vida Familiar — Adaptada 2021

Personas con enfermedades neurodegenerativas.

¿Qué es la Escala de Calidad de Vida Familiar?

La Escala de Calidad de Vida Familiar (Escala FQOL) es un método para centrarse en la calidad de vida de las familias que tienen uno o más miembros con enfermedad neurodegenerativa. Es una manera de abordar el grado en que la calidad de vida de la familia es agradable, significativa y apoyada por los tipos de recursos que son importantes para los miembros de la familia, así como las dificultades que las familias afrontan.

¿Cómo se debe utilizar la Escala FQOL?

La escala FQOL está destinada a dos usos. En primer lugar, puede ser útil para los profesionales del servicio y los miembros de la familia como parte de una evaluación general de las necesidades de apoyo y de diseño del programa. En segundo lugar, se puede utilizar como instrumento para describir y medir la calidad de vida familiar, dentro de las limitaciones de su conceptualización, con fines de investigación o de evaluación.

Estructura de la Escala FQOL

Hay muchas maneras de ver la calidad de vida familiar. La Escala FQOL analiza aspectos de la vida familiar que consideramos críticos, basado en investigaciones y prácticas anteriores.

La Escala FQOL tiene varias partes:

La primera parte, Acerca de su familia, presenta a los miembros de su familia.

Las siguientes 9 partes abordan áreas específicas de la vida familiar: salud, bienestar financiero, relaciones familiares, apoyo de los demás, apoyo de los servicios, influencia de los valores, carreras, ocio y recreación, e integración comunitaria. Cada una de estas 9 partes tiene 2 secciones. La sección A contiene preguntas que recopilan cierta información general y proporcionan un contexto. La sección B contiene preguntas relacionadas con 6 conceptos clave: importancia, oportunidades, iniciativa, logro, estabilidad y satisfacción.

Estas preguntas pueden parecer algo repetitivas. Están destinadas a ser así, porque hacen la misma pregunta sobre cada una de las 9 áreas diferentes de la vida.

La parte corta final de la Escala FQOL pide impresiones generales de la calidad de vida familiar.

¿Cómo se debe administrar la Escala FQOL?

La Escala FQOL puede ser completada por el cuidador principal (autoadministración) o por un investigador o personal clínico con el cuidador principal (cara a cara, o administración de entrevistas telefónicas). Cuando sea autoadministrada, se debe

facilitar a la persona que conteste el cuestionario una manera de contactar con alguien, que conozca la Escala, para que le resuelva las dudas.

El consentimiento informado de la persona que responde a la Escala siempre debe obtenerse por escrito.

En algunos casos, los investigadores consideran que es útil aumentar la información recopilada por la Escala con una entrevista personal.

Confidencialidad y Consideraciones Éticas

La Escala completa de FQOL contiene información confidencial. Cuando se utilice por organizaciones de servicios para la evaluación de las necesidades de apoyo y el diseño de programas, se deben utilizar las políticas y directrices establecidas para toda la información confidencial. Cuando la Escala FQOL se utiliza con fines de investigación o evaluación, toda la información personal de las familias y los individuos con demencia debe ser ocultada, y solo se deben reportar los datos agregados o ejemplos de casos anónimos. Quienes utilicen esta encuesta deben cumplir con los requisitos éticos de sus universidades, organizaciones u órganos de gobierno pertinentes.

Enfermedades neurodegenerativas

Las enfermedades neurodegenerativas se caracterizan por la muerte de neuronas en diferentes regiones del sistema nervioso y el consiguiente deterioro funcional de las partes afectadas. Esta pérdida progresiva de las células nerviosas es lo que origina los signos y síntomas neurológicos y neuropsicológicos característicos de cada una de ellas. Todas presentan como característica su cronicidad y su evolución progresiva. Son enfermedades que no solo afectan a quien las sufre sino también a su entorno más cercano. En general, provocan alteraciones en muchas actividades y funciones corporales como es el equilibrio, la movilidad, el habla, la respiración y la función cardíaca, entre otras. Así, lesionan áreas físicas vinculadas con la esencia humana: la memoria, la percepción, los sentimientos, la capacidad de tomar decisiones. Aspectos tan vitales de la vida humana que generan efectos muy devastadores y un especial sufrimiento. http://neuroalianza.org/las-enfermedades-neurodegenerativas/que-son/

Traducción y adaptación

La *Escala de Calidad de Vida Familiar: Personas con Enfermedades Neurodegenerativas* es una versión traducida y adaptada al español de *Escala de Calidad de Vida Familiar: Cuidadores Principales de personas con Demencia*, que se desarrolló en inglés. Es nuestro deseo traducir la escala a otros idiomas, y tener las versiones traducidas disponibles en nuestra página Web. Si quisiera traducir la escala a su idioma para usarla en su país, le agradeceríamos que contactara con nosotros para que podamos trabajar juntos para que esté disponible.

¿Tiene dificultades al descargar la Escala de CVF?

Si encuentra dificultades al descargar la escala o para obtener una copia con el formato adecuado, por favor contacte con Barry Isaacs: barry.isaacs@surreyplace.on.ca.

Instrucciones para completar la Escala de CVF

¿Quién responde las preguntas de la escala?

La Escala de Calidad de Vida Familiar será completada por el cuidador principal del familiar o por la propia persona con enfermedad neurodegenerativa. El cuidador principal puede ser el/la padre/madre, un hermano/a, el cónyuge o pareja, u otro miembro de la familia tal como se define en la sección "Acerca de su familia" (página 1). Puede ser completada por el miembro de la familia con enfermedad neurodegenerativa o por un miembro de la familia que puede ayudar en la atención.

Al completar esta escala

1. Por favor responda cada pregunta de la forma más completa posible.
2. Completar la escala lleva alrededor de una hora.

Cuestionario sociodemográfico: entrevistado

<table>
<tr><td>Indique si es el cuidador principal de la persona con Enfermedad Neurodegenerativa</td><td>Si □ 1</td><td colspan="2">No □ 2</td></tr>
<tr><td>Persona con enfermedad neurodegenerativa</td><td>Si □ 1</td><td colspan="2">No □ 2</td></tr>
<tr><td>Edad</td><td colspan="3"></td></tr>
<tr><td>Sexo</td><td>Masculino □ 1</td><td colspan="2">Femenino □ 2</td></tr>
<tr><td rowspan="2">Nivel de estudios</td><td>Primarios □ 1</td><td colspan="2">Secundarios □ 2</td></tr>
<tr><td>Superiores □ 3</td><td>Otros □ 4</td><td>Sin estudios □ 0</td></tr>
<tr><td rowspan="2">Situación laboral</td><td>Empleado □ 1</td><td colspan="2">Pensionista □ 2</td></tr>
<tr><td>Desempleado □ 3</td><td>Otros □ 5</td><td>Autónomo □ 4</td></tr>
<tr><td rowspan="3">Nivel de ingresos mensuales</td><td>Menos 500 € □ 1</td><td colspan="2">Entre 500–1.000 € □ 2</td></tr>
<tr><td>Entre 1.000–1.500 € □ 3</td><td colspan="2">Entre 1.500–2.000 € □ 4</td></tr>
<tr><td colspan="3">Más de 2.000 € □ 5</td></tr>
<tr><td rowspan="2">Estado civil</td><td>En pareja □ 1</td><td colspan="2">Separado/a □ 2</td></tr>
<tr><td>Viudo/a □ 3</td><td colspan="2">Soltero/a □ 4</td></tr>
</table>

Sobre su familia

En el mundo existen diferentes maneras de entender lo que es una familia. En este cuestionario le pedimos que piense en su familia; es decir, aquellas personas que están muy implicadas en los asuntos cotidianos de su casa. Los miembros de la familia pueden estar unidos por consanguinidad o por una relación personal cercana.

1. ¿Cuál es su relación con el familiar (o familiares) con enfermedad neurodegenerativa? Soy su…

Persona 1	Pareja □ 1	Hijo □ 2	Hija □ 3	Nuera □ 4
	Yerno □ 5	Hermano □ 6	Hermana □ 7	Nieto □ 8
	Nieta □ 9	Padre □ 10	Madre □ 11	Otra (especificar): 12

Persona 2	Pareja □ 1	Hijo □ 2	Hija □ 3	Nuera □ 4
	Yerno □ 5	Hermano □ 6	Hermana □ 7	Nieto □ 8
	Nieta □ 9	Padre □ 10	Madre □ 11	Otra (especificar): 12

2. Por favor, indique el género y la edad de todos los miembros de la familia con una enfermedad neurodegenerativa:

	Género	*Edad*	*Mismo domicilio*
Persona 1	Hombre □ 1		Si □ 1
	Mujer □ 2		No □ 2
Persona 2	Hombre □ 1		Si □ 1
	Mujer □ 2		No □ 2

3. Por favor, indique la situación de dependencia de todos los miembros de la familia con una enfermedad neurodegenerativa:

	Reconocimiento	*Grado de dependencia*		
Persona 1	Si □ 1	Grado 1 □ 1	Grado 2 □ 2	Grado 3 □ 3
	No □ 2			
Persona 2	Si □ 1	Grado 1 □ 1	Grado 2 □ 2	Grado 3 □ 3
	No □ 2			

4a. A continuación, se indican algunos diagnósticos que pueden estar relacionados con la enfermedad neurodegenerativa. Por favor, marque ✓ todos los que afectan a alguno/s de sus familiares:

Persona 1	*Persona 2*	
Si □ 1<br>No □ 2	Si □ 1<br>No □ 2	Alzheimer
Si □ 1<br>No □ 2	Si □ 1<br>No □ 2	Demencia vascular
Si □ 1<br>No □ 2	Si □ 1<br>No □ 2	Demencia mixta
Si □ 1<br>No □ 2	Si □ 1<br>No □ 2	Demencia frontotemporal

Si □ 1<br>No □ 2	Si □ 1<br>No □ 2	Demencia con cuerpos de Lewy
Si □ 1<br>No □ 2	Si □ 1<br>No □ 2	Parkinson
Si □ 1<br>No □ 2	Si □ 1<br>No □ 2	Esclerosis múltiple
Si □ 1<br>No □ 2	Si □ 1<br>No □ 2	Otra/s (especificar):

4b. Las personas con enfermedad neurodegenerativa a menudo tienen otros problemas asociados (Marque ✓ los que afectan a su/s familiar/es).

Persona 1	*Persona 2*	
Si □ 1<br>No □ 2	Si □ 1<br>No □ 2	Cambios de conducta y carácter (desinhibición, agresividad)
Si □ 1<br>No □ 2	Si □ 1<br>No □ 2	Depresión, ansiedad y otros trastornos psiquiátricos
Si □ 1<br>No □ 2	Si □ 1<br>No □ 2	Problemas de coordinación motriz
Si □ 1<br>No □ 2	Si □ 1<br>No □ 2	Convulsiones
Si □ 1<br>No □ 2	Si □ 1<br>No □ 2	Pérdida de memoria
Si □ 1<br>No □ 2	Si □ 1<br>No □ 2	Dificultades del habla y/o lenguaje
Si □ 1<br>No □ 2	Si □ 1<br>No □ 2	Alcoholismo
Si □ 1<br>No □ 2	Si □ 1<br>No □ 2	Otros problemas de salud
Si □ 1<br>No □ 2	Si □ 1<br>No □ 2	Otro/s (especificar):

5. **¿Qué apoyos relacionados con la enfermedad neurodegenerativa necesita/n su/s familiar/es para las distintas áreas de la vida?** (Marque ✓ una opción por cada familiar con enfermedad neurodegenerativa).

Persona 1	*Persona 2*	
5	5	No requiere
4	4	Muy pocos apoyos
3	3	Algunos apoyos
2	2	Bastantes apoyos
1	1	Muchos apoyos

6. **¿Qué capacidad de comunicación tiene/n su/s familiar/es con enfermedad neurodegenerativa?** (Marque ✓ **una opción** por cada familiar con enfermedad neurodegenerativa).

Persona 1	*Persona 2*	
5	5	Capaz de comunicarse de forma coherente sobre muchos temas

4	4	Capaz de comunicarse de forma coherente sobre pocos temas
3	3	Capaz de comunicar necesidades, deseos y algunas ideas de forma coherente
2	2	Capaz de comunicar solo necesidades básicas y deseos
1	1	Muy poca comunicación coherente

7. **Por favor describa el grado de independencia de su/s familiar/es con enfermedad neurodegenerativa en las siguientes actividades básicas de la vida diaria.**

A. Alimentación: uso de utensilios para llevar alimentos y bebida a la boca y masticarlos/tragarlos.

Persona 1	*Persona 2*	
5	5	No requiere ayuda
4	4	Muy poca ayuda
3	3	Algo de ayuda
2	2	Bastante ayuda
1	1	Mucha ayuda

B. Baño/ducha: lavar, aclarar y secar partes de su cuerpo.

Persona 1	*Persona 2*	
5	5	No requiere ayuda
4	4	Muy poca ayuda
3	3	Algo de ayuda
2	2	Bastante ayuda
1	1	Mucha ayuda

C. Aseo y cuidado personal: peinarse, lavarse la cara, afeitarse, maquillarse, cepillarse los dientes, enjuagarse la boca.

Persona 1	*Persona 2*	
5	5	No requiere ayuda
4	4	Muy poca ayuda
3	3	Algo de ayuda
2	2	Bastante ayuda
1	1	Mucha ayuda

D. Vestido: vestirse y desvestirse.

Persona 1	*Persona 2*	
5	5	No requiere ayuda
4	4	Muy poca ayuda
3	3	Algo de ayuda
2	2	Bastante ayuda
1	1	Mucha ayuda

E. Uso del inodoro: utilizar el WC, limpiar la zona perianal y ajustarse la ropa.

Persona 1	*Persona 2*	
5	5	No requiere ayuda
4	4	Muy poca ayuda
3	3	Algo de ayuda
2	2	Bastante ayuda
1	1	Mucha ayuda

F. Movilidad en el hogar: sentarse estando tumbado, levantarse estando sentado, y moverse desde una silla de ruedas a la cama/silla/bañera e inodoro.

Persona 1	*Persona 2*	
5	5	No requiere ayuda
4	4	Muy poca ayuda
3	3	Algo de ayuda
2	2	Bastante ayuda
1	1	Mucha ayuda

8. **Por favor, describa el grado de autonomía de su/s familiar/es con enfermedad neurodegenerativa en las siguientes actividades instrumentales de la vida diaria**.

A. Tareas domésticas: limpiar el polvo, aspirar/barrer, organizar objetos domésticos tales como platos o ropa, hacer la cama, lavar la ropa y sacar la basura.

Persona 1	*Persona 2*	
5	5	No requiere ayuda
4	4	Muy poca ayuda
3	3	Algo de ayuda
2	2	Bastante ayuda
1	1	Mucha ayuda

B. Realización de compras: hacer la lista de la compra, comprar alimentos, ropa y otros objetos personales.

Persona 1	*Persona 2*	
5	5	No requiere ayuda
4	4	Muy poca ayuda
3	3	Algo de ayuda
2	2	Bastante ayuda
1	1	Mucha ayuda

C. Gestión del dinero: administrar el dinero para compras, revisar sus finanzas y mantener cuentas bancarias.

Persona 1	*Persona 2*	
5	5	No requiere ayuda

4	4	Muy poca ayuda
3	3	Algo de ayuda
2	2	Bastante ayuda
1	1	Mucha ayuda

D. Preparación de la comida: puede planificar y preparar comidas frías y calientes, y ocuparse de cortar, cocinar y servir.

Persona 1	*Persona 2*	
5	5	No requiere ayuda
4	4	Muy poca ayuda
3	3	Algo de ayuda
2	2	Bastante ayuda
1	1	Mucha ayuda

E. Desplazamiento fuera del hogar: puede caminar y utilizar transporte público (por ejemplo: autobús, coche compartido, taxi) o privado (por ejemplo: conducir, andar en bicicleta).

Persona 1	*Persona 2*	
5	5	No requiere ayuda
4	4	Muy poca ayuda
3	3	Algo de ayuda
2	2	Bastante ayuda
1	1	Mucha ayuda

9. **En este momento ¿cuántas personas viven con usted en casa de forma habitual, incluida usted? _______**

10. **Qué miembro/s de la familia está/n más implicado/s en la vida cotidiana de su/s familiar/es con enfermedad neurodegenerativa?** (Marque ✓ todas las opciones que correspondan).

La pareja del familiar con enfermedad neurodegenerativa	Si □ 1<br>No □ 2
El/los hijo/s	Si □ 1<br>No □ 2
La/s hija/s	Si □ 1<br>No □ 2
El/los yernos	Si □ 1<br>No □ 2
La/s nueras	Si □ 1<br>No □ 2
El/los hermanos	Si □ 1<br>No □ 2
La/s hermanas	Si □ 1<br>No □ 2
El/los nietos	Si □ 1<br>No □ 2

La/s nietas	Si □ 1<br>No □ 2
Padre	Si □ 1<br>No □ 2
Madre	Si □ 1<br>No □ 2
Otros(especificar):	
Otros (especificar)	

11. **¿Cómo valora la responsabilidad que tiene usted en el cuidado de su/s familiar/es con enfermedad neurodegenerativa**? (Marque ✓ una opción).

1 Mucha más responsabilidad de la que me gustaría
2 Más de la que me gustaría
3 Más o menos la que me gustaría
4 Menos de la que me gustaría
5 Mucha menos de la que me gustaría

12. **¿Cómo valora la responsabilidad que tiene usted en los asuntos cotidianos de su familia** (por ejemplo: hacer la compra, economía familiar, etc.)? (Marque ✓ una opción).

1 Mucha más responsabilidad de la que me gustaría
2 Más de la que me gustaría
3 Más o menos la que me gustaría
4 Menos de la que me gustaría
5 Mucha menos de la que me gustaría

1. La salud de la familia

En esta sección le pedimos que valore el **estado general de salud de su familia**. En ocasiones, uno o varios miembros de una familia tienen problemas de salud y ello afecta a los otros miembros. Cuando responda las siguientes preguntas, **piense en su familia**.

Sección A

1. ¿Cómo es el estado de salud física de su/s familiar/es con enfermedad neurodegenerativa en la actualidad?

	Persona 1	*Persona 2*
Excelente	5	5
Muy bueno	4	4
Bueno	3	3
Regular	2	2
Malo	1	1

2. ¿Cómo es su propio estado de salud en la actualidad?

Excelente	5
Muy bueno	4
Bueno	3
Regular	2
Malo	1

3. ¿Otros miembros de su familia tienen problemas graves de salud física y/o mental?

1	Sí
2	No

4. ¿Qué obstáculos encuentra su familia para recibir la atención sanitaria? (Marque ✓ tantas opciones como proceda).

1	Largos tiempos de espera
2	No hay servicios sanitarios disponibles en nuestra zona
3	Hay problemas de transporte
4	No podemos pagar el coste de algunos servicios
5	No sabemos a donde acudir para recibir servicios de atención sanitaria
6	Nos cuesta entender lo que dicen los profesionales sanitarios
7	El trato que recibimos de los profesionales sanitarios es de mala calidad
8	Tenemos diferentes opiniones sobre la atención sanitaria
	Otro (especifica cuál):

Sección B

1. **¿Cuánta importancia tiene la salud para la calidad de vida de su familia?**

5	Mucha importancia
4	Bastante importancia
3	Algo de importancia
2	Poca importancia
1	Casi sin importancia

2. **¿Hay oportunidades en su zona para cubrir las necesidades de salud de su familia?**

5	Muchas
4	Bastantes
3	Algunas
2	Muy pocas
1	Ninguna

3. **¿Los miembros de su familia se esfuerzan por mantener y/o mejorar su salud** (por ejemplo: haciendo ejercicio de manera regular o cuidando su dieta)?

5	Mucho
4	Bastante
3	Algo
2	Muy poco
1	Nada

4. **¿Los miembros de su familia tienen buena salud?**

5	Mucha
4	Bastante
3	Algo
2	Muy poca
1	Nada

5. **En un futuro próximo, es probable que el nivel de salud de su familia…**

5	Mejore mucho
4	Mejore
3	Sea similar
2	Empeore
1	Empeore mucho

6. **¿En qué medida su familia está satisfecha con su salud?**

5	Muy satisfecha
4	Satisfecha

3 Ni satisfecha ni insatisfecha
2 Insatisfecha
1 Muy insatisfecha

2. Bienestar económico

En esta sección le pedimos que piense cómo gestiona su familia los asuntos económicos. Aunque los distintos miembros tengan diferentes ingresos y necesidades económicas, piense en la situación económica del conjunto de su familia cuando responda a las siguientes preguntas.

Sección A

1. **Cuando usted piensa en los ingresos totales de la familia, incluyendo todas las pensiones, considera que su familia está** (marque ✓ una opción):

5 Muy bien
4 Nos manejamos bien, con algún extra
3 Bien
2 Nos arreglamos
1 Con dificultades

2. **¿Qué ingresos dedican los miembros de la familia (incluidas las pensiones y prestaciones), por término medio, en cuidados especiales, medicamentos, apoyo o equipamiento para el/los familiar/es con enfermedades neurodegenerativas?** (Marque ✓ una opción).

1 Ninguno
2 Menos de la mitad
3 La mitad
4 Más de la mitad

3. **¿Cuántas necesidades básicas de su familia (por ejemplo: comida, ropa, vivienda) se cubren con los ingresos familiares?** (Marque ✓ una opción).

5 Todas
4 La mayoría
3 Algunas
2 Muy pocas
1 Ninguna

4. **Después de pagar todos los gastos necesarios, ¿al final de mes le queda dinero a su familia para hacer las cosas que desea?** (Marque ✓ una opción).

1 Sí
2 No

Sección B

1. **¿Cuánta importancia tiene el bienestar económico para la calidad de vida de su familia?**

   - 5 Mucha importancia
   - 4 Bastante importancia
   - 3 Algo de importancia
   - 2 Poca importancia
   - 1 Casi sin importancia

2. **¿Hay oportunidades para que los miembros de su familia ganen dinero suficiente para hacer las cosas que desean?**

   - 5 Muchas
   - 4 Bastantes
   - 3 Algunas
   - 2 Muy pocas
   - 1 Ninguna

3. **¿Se esfuerzan los miembros de su familia para mantener y/o mejorar su situación económica?**

   - 5 Mucho
   - 4 Bastante
   - 3 Algo
   - 2 Muy poco
   - 1 Nada

4. **¿En qué medida la situación económica de su familia responde a lo que usted esperaba?**

   - 5 Mucho
   - 4 Bastante
   - 3 Algo
   - 2 Muy poco
   - 1 Nada

5. **En un futuro próximo, es probable que la situación económica de su familia…**

   - 5 Mejore mucho
   - 4 Mejore
   - 3 Sea similar
   - 2 Empeore
   - 1 Empeore mucho
   - No sabe, no contesta

6. **¿En qué medida su familia está satisfecha con la situación económica?**

5	Muy satisfecha
4	Satisfecha
3	Ni satisfecha ni insatisfecha
2	Insatisfecha
1	Muy insatisfecha

3. Relaciones familiares

En esta sección, piense en el tono o sentimiento general que está presente de forma habitual en su familia. Una persona puede llevarse mejor con unos familiares que con otros, pero aquí nos interesa conocer el **ambiente que reina en su familia.**

Sección A

1. **En general, en qué medida en su familia…** (Marque ✓ una respuesta para cada enunciado).

	Mucho	*Bastante*	*Algo*	*Poco*	*Nada*
a) Se ayudan a hacer las cosas	1	2	3	4	5
b) Van juntos a los sitios	1	2	3	4	5
c) Disfrutan de la compañía de los otros	1	2	3	4	5
d) Se apoyan en los momentos difíciles	1	2	3	4	5
e) Se ayudan a solucionar problemas familiares	1	2	3	4	5
f) Confían unos en los otros	1	2	3	4	5
g) Trabajan juntos para lograr metas comunes	1	2	3	4	5
h) Tienen un sentimiento de pertenencia	1	2	3	4	5
i) Tienen valores similares	1	2	3	4	5
j) Hacen planes familiares	1	2	3	4	5

Sección B

1. **¿Cuánta importancia tienen las relaciones familiares para la calidad de vida de su familia?**

5	Mucha importancia
4	Bastante importancia
3	Algo de importancia
2	Poca importancia
1	Casi sin importancia

2. **¿Los miembros de su familia disponen de oportunidades para mantener y mejorar las buenas relaciones entre ellos?**

5	Muchas
4	Bastantes
3	Algunas

2 Muy pocas
1 Ninguna

3. **¿Se esfuerzan los miembros de su familia por mantener buenas relaciones entre ellos?**

5 Mucho
4 Bastante
3 Algo
2 Muy poco
1 Nada

4. **¿En qué medida los miembros de su familia disfrutan de buenas relaciones entre ellos?**

5 Mucha
4 Bastante
3 Algo
2 Muy poca
1 Nada

5. **En un futuro próximo, es probable que las relaciones familiares…**

5 Mejoren mucho
4 Mejoren
3 Sean similares
2 Empeoren
1 Empeoren mucho
☐ No sabe, no contesta

6. **¿En qué medida su familia está satisfecha de las relaciones familiares?**

5 Muy satisfecha
4 Satisfecha
3 Ni satisfecha ni insatisfecha
2 Insatisfecha
1 Muy insatisfecha

4. Apoyo de otras personas

A veces, las familias reciben apoyo práctico y emocional de diversas personas, como, por ejemplo, parientes, amigos, vecinos y otros. En esta sección le pedimos que piense en el **apoyo que recibe su familia de otras personas**.

Sección A

1. **¿Cuánto les ayudan sus parientes (distintos de aquellos que usted ha identificado como su familia) en asuntos prácticos, como cuidar de miembros de la familia, comprar o cuidar la casa?** (Marque ✓ una opción).

5	Mucho
4	Bastante
3	Algo
2	Muy poco
1	Nada

2. **¿Cuánto apoyo emocional reciben de sus parientes (distintos de aquellos que usted ha identificado como su familia), por ejemplo, hablando con ustedes, escuchándolos o dándoles ánimos?** (Marque ✓ una opción).

5	Mucho
4	Bastante
3	Algo
2	Muy poco
1	Nada

3. **¿Cuánto les ayudan sus amigos y vecinos en asuntos prácticos, como cuidar de miembros de la familia, comprar o cuidar la casa?** (Marque ✓ una opción).

5	Mucho
4	Bastante
3	Algo
2	Muy poco
1	Nada

4. **¿Cuánto apoyo emocional reciben de sus amigos y vecinos, por ejemplo, hablando con ustedes, escuchándolos o dándoles ánimos?** (Marque ✓ una opción).

5	Mucho
4	Bastante
3	Algo
2	Muy poco
1	Nada

5. **¿Cómo describiría su vida social al margen de su familia?** (Marque ✓ una opción).

5	Muy buena
4	Buena
3	Regular
2	Mala
1	Muy mala

Sección B

1. **¿Cuánta importancia tiene para la calidad de vida de su familia el apoyo práctico que recibe de otras personas, excluyendo los servicios profesionales?**

   - 5 Mucha importancia
   - 4 Bastante importancia
   - 3 Algo de importancia
   - 2 Poca importancia
   - 1 Casi sin importancia

2. **¿Cuánta importancia tiene para la calidad de vida de su familia el apoyo emocional que recibe de otras personas, excluyendo los servicios profesionales?** (por ejemplo: compañía, consuelo, comprensión, etc.).

   - 5 Mucha importancia
   - 4 Bastante importancia
   - 3 Algo de importancia
   - 2 Poca importancia
   - 1 Casi sin importancia

3. **¿Hay oportunidades para que los miembros de su familia reciban apoyo práctico de otras personas, excluyendo los servicios profesionales?**

   - 5 Muchas
   - 4 Bastantes
   - 3 Algunas
   - 2 Muy pocas
   - 1 Ninguna

4. **¿Hay oportunidades para que los miembros de su familia reciban apoyo emocional de otras personas, excluyendo los servicios profesionales?** (por ejemplo: compañía, consuelo, comprensión, etc.)

   - 5 Muchas
   - 4 Bastantes
   - 3 Algunas
   - 2 Muy pocas
   - 1 Ninguna

5. **¿Se esfuerzan los miembros de su familia para conseguir apoyo práctico de otras personas, excluyendo los servicios profesionales?**

   - 5 Mucho
   - 4 Bastante
   - 3 Algo
   - 2 Muy poco
   - 1 Nada

6. ¿Se esfuerzan los miembros de su familia para conseguir apoyo emocional de otras personas, excluyendo los servicios profesionales?

5 Mucho
4 Bastante
3 Algo
2 Muy poco
1 Nada

7. ¿En qué medida su familia recibe apoyo práctico de otras personas, excluyendo los servicios profesionales?

5 Mucho
4 Bastante
3 Algo
2 Muy poco
1 Nada

8. ¿En qué medida su familia recibe apoyo emocional de otras personas, excluyendo los servicios profesionales?

5 Mucho
4 Bastante
3 Algo
2 Muy poco
1 Nada

9. En el futuro próximo, es probable que el apoyo práctico que reciba de otras personas, excluyendo los servicios profesionales, …

5 Mejore mucho
4 Mejore
3 Sea similar
2 Empeore
1 Empeore mucho

10. En el futuro próximo, es probable que el apoyo emocional que reciba de otras personas, excluyendo los servicios profesionales, …

5 Mejore mucho
4 Mejore
3 Sea similar
2 Empeore
1 Empeore mucho

11. ¿En qué medida está satisfecho de la ayuda práctica que recibe su familia de otras personas, excluyendo los servicios profesionales?

5	Muy satisfecho
4	Satisfecho
3	Ni satisfecho ni insatisfecho
2	Insatisfecho
1	Muy insatisfecho

12. **¿En qué medida está satisfecho de la ayuda emocional que recibe su familia de otras personas, excluyendo los servicios profesionales?**

5	Muy satisfecho
4	Satisfecho
3	Ni satisfecho ni insatisfecho
2	Insatisfecho
1	Muy insatisfecho

5. Apoyo de los servicios de atención a personas con enfermedades neurodegenerativas

En esta sección le pedimos que piense en el apoyo que recibe de los servicios de atención a personas con enfermedad neurodegenerativa. Aunque estos servicios suelen destinarse directamente a la persona con enfermedad neurodegenerativa, a menudo afectan a la familia en su conjunto.

Sección A

1. **¿Cuál/es de los siguientes servicios ha usado usted o algún miembro de su familia desde que su/s familiar/es recibió el diagnóstico?** (Marque ✓ todas las opciones que proceda).

Si □ 1<br>No □ 2	Prestaciones y ayudas económicas
Si □ 1<br>No □ 2	Prestaciones sociales básicas (por ejemplo: teleasistencia, ayuda a domicilio)
Si □ 1<br>No □ 2	Centros de acción social (CEAS)
Si □ 1<br>No □ 2	Cuidador contratado privado
Si □ 1<br>No □ 2	Servicio de respiro (por ejemplo: residencias temporales, centros de día, centros de rehabilitación)
Si □ 1<br>No □ 2	Asesoramiento jurídico
Si □ 1<br>No □ 2	Programas de convivencia y participación social (por ejemplo: programas de empleo protegido, taller de memoria, taller de actividad física, programa de apoyo a la vida independiente, etc.)
Si □ 1<br>No □ 2	Servicio de proximidad (por ejemplo: comida a domicilio, servicios de lavandería, etc.)
Si □ 1<br>No □ 2	Atención sanitaria básica (por ejemplo: médico de familia, atención a domicilio, etc.)

Si □ 1<br>No □ 2	Atención sanitaria especializada (por ejemplo: neurólogo, psiquiatra, etc.)
Si □ 1<br>No □ 2	Asociaciones (por ejemplo: Alzheimer, Esclerosis Múltiple, Parkinson, etc.)
Si □ 1<br>No □ 2	Voluntariado
Si □ 1<br>No □ 2	Otro/s (por favor, especifique cuál/es):

2. **¿Hay servicios de atención a personas con enfermedades neurodegenerativas que necesite la familia, pero no los esté recibiendo actualmente?**

□ 1	Sí
□ 2	No (pase a la ***Sección B***)
□ 3	No sé

3. **Si ha contestado Sí a la pregunta anterior, ¿por qué no está recibiendo estos servicios?** (Marque ✓ todas las opciones que proceda).

Si □ 1<br>No □ 2	Largos tiempos de espera
Si □ 1<br>No □ 2	No hay servicios disponibles en mi zona
Si □ 1<br>No □ 2	Problemas de transporte
Si □ 1<br>No □ 2	Tenemos problemas para desplazarnos
Si □ 1<br>No □ 2	No podemos conseguir una cita con facilidad
Si □ 1<br>No □ 2	No sabemos a donde acudir para recibir estos servicios
Si □ 1<br>No □ 2	Nos cuesta entender lo que nos dicen los profesionales de estos servicios
Si □ 1<br>No □ 2	No podemos hacer frente a los costes de algunos de estos servicios
Si □ 1<br>No □ 2	No disponemos de apoyos para acudir a estos servicios

Sección B

1. **¿Cuánta importancia tiene para la calidad de vida de su familia la ayuda que recibe de los servicios sociales y de atención médica a personas con enfermedades neurodegenerativas?**

5	Mucha importancia
4	Bastante importancia
3	Algo de importancia
2	Poca importancia

1 Casi sin importancia

2. ¿Hay oportunidades en su zona para recibir los servicios sociales y de atención médica a personas con enfermedades neurodegenerativas que necesita su familia?

5 Muchas
4 Bastantes
3 Algunas
2 Muy pocas
1 Ninguna

3. ¿Se esfuerzan los miembros de su familia para obtener los servicios sociales y de atención médica a personas con enfermedades neurodegenerativas que necesitan?

5 Mucho
4 Bastante
3 Algo
2 Muy poco
1 Nada

4. ¿En qué medida son atendidas en su zona las necesidades de su familia, relacionadas con la/s persona/s con enfermedad neurodegenerativa?

5 Mucho
4 Bastante
3 Algo
2 Muy poco
1 Nada

5. En un futuro próximo, es probable que la ayuda que reciba su familia de los servicios sociales y de atención médica a personas con enfermedades neurodegenerativas...

5 Mejore mucho
4 Mejore
3 Sea similar
2 Empeore
1 Empeore mucho

6. ¿En qué medida su familia está satisfecha con los servicios sociales y de atención médica a personas con enfermedades neurodegenerativas?

5 Muy satisfecha
4 Satisfecha
3 Ni satisfecha ni insatisfecha
2 Insatisfecha
1 Muy insatisfecha

6. Influencia de los valores

Muchas personas obtienen satisfacción de sus valores. Esos valores proceden de creencias personales, espirituales, religiosas y culturales. Para la mayoría, los valores surgen de una combinación de estas creencias. En esta sección le pedimos que piense en el grado en que su familia se ve afectada por valores personales, espirituales, religiosos y culturales, considerando el impacto sobre su familia en su conjunto.

Sección A

1. **Los valores de su familia son…** (Marque ✓ todas las opciones que proceda).

   1 Personales
   2 Espirituales
   3 Religiosos
   4 Culturales

2. **¿En qué medida acepta su comunidad religiosa, espiritual o cultural a la persona con enfermedad neurodegenerativa?**

   5 Mucho
   4 Bastante
   3 Algo
   2 Muy poco
   1 Nada

3. **¿En qué medida las personas de su comunidad religiosa, espiritual o cultural ayudan de forma práctica (como cuidar de miembros de la familia, comprar o cuidar la casa) a su familia en sus necesidades relacionadas con la enfermedad neurodegenerativa?**

   5 Mucho
   4 Bastante
   3 Algo
   2 Muy poco
   1 Nada

4. **¿En qué medida las personas de su comunidad religiosa, espiritual o cultural apoyan emocionalmente (hablando con ustedes, escuchándolos o dándoles ánimos) a su familia en sus necesidades relacionadas con la persona con enfermedad neurodegenerativa?**

   5 Mucho
   4 Bastante
   3 Algo
   2 Muy poco
   1 Nada

5. ¿En qué medida sus valores personales, espirituales, religiosos y/o culturales ayudan a los miembros de su familia a aceptar y afrontar la enfermedad neurodegenerativa?

5	Mucho
4	Bastante
3	Algo
2	Muy poco
1	Nada

Sección B

1. ¿Cuánta importancia tienen los valores personales, espirituales, religiosos y/o culturales para la calidad de vida de su familia?

5	Mucha importancia
4	Bastante importancia
3	Algo de importancia
2	Poca importancia
1	Casi sin importancia

2. ¿Hay oportunidades para que los miembros de su familia desarrollen y/o mantengan valores personales, espirituales, religiosos y/o culturales que contribuyan a la calidad de vida de su familia?

5	Muchas
4	Bastantes
3	Algunas
2	Muy pocas
1	Ninguna

3. ¿Se esfuerzan los miembros de su familia para mantener y/ o reforzar valores personales, espirituales, religiosos y/o culturales que contribuyan a la calidad de vida de su familia?

5	Mucho
4	Bastante
3	Algo
2	Muy poco
1	Nada

4. ¿En qué medida los miembros de su familia mantienen valores personales, espirituales, religiosos y/o culturales que contribuyen a la calidad de vida de su familia?

5	Mucho
4	Bastante
3	Algo
2	Muy poco

1	Nada

5. **En un futuro próximo, es probable que los valores personales, espirituales, religiosos y/o culturales que contribuyen a la calidad de vida de su familia….**

5	Mejoren mucho
4	Mejoren
3	Sean similares
2	Empeoren
1	Empeoren mucho

6. **¿En qué medida los miembros de su familia están satisfechos con los valores personales, espirituales, religiosos y/o culturales que contribuyen a la calidad de vida de su familia**

5	Muy satisfechos
4	Satisfechos
3	Ni satisfechos ni insatisfechos
2	Insatisfechos
1	Muy insatisfechos

7. Profesiones y formación de los cuidadores

Parte de la vida de un adulto implica desarrollar una profesión. De manera similar, parte de la vida de un menor supone formarse para el futuro. En esta sección piense en su familia en su conjunto.

Sección A

1. **¿Qué actividades laborales y/o formativas lleva a cabo el miembro (o los miembros) con enfermedad neurodegenerativa?** (Marque ✓ todas las opciones que proceda para cada familiar).

Persona 1	*Persona 2*	
Si □ 1<br>No □ 2	Si □ 1<br>No □ 2	Ninguna
Si □ 1<br>No □ 2	Si □ 1<br>No □ 2	Empleo ordinario
Si □ 1<br>No □ 2	Si □ 1<br>No □ 2	Empleo con apoyo
Si □ 1<br>No □ 2	Si □ 1<br>No □ 2	Autónomo
Si □ 1<br>No □ 2	Si □ 1<br>No □ 2	Voluntario
Si □ 1<br>No □ 2	Si □ 1<br>No □ 2	Centro Especial de Empleo
Si □ 1<br>No □ 2	Si □ 1<br>No □ 2	Formación profesional
Si □ 1	Si □ 1	Educación básica

No □ 2	No □ 2	
Si □ 1<br>No □ 2	Si □ 1<br>No □ 2	Educación superior

2. **¿Qué miembro/s de tu familia han abandonado sus estudios o su profesión para cuidar del familiar/es con enfermedad neurodegenerativa?** (Marque ✓ tantas opciones como proceda).

Ninguno/a □<br>Si □ 1 No □ 2			
La pareja □<br>Si □ 1 No □ 2	El hijo □<br>Si □ 1 No □ 2	La hija □<br>Si □ 1 No □ 2	La nuera □<br>Si □ 1 No □ 2
El yerno □<br>Si □ 1 No □ 2	El hermano □<br>Si □ 1 No □ 2	La hermana □<br>Si □ 1 No □ 2	El nieto □<br>Si □ 1 No □ 2
La nieta □<br>Si □ 1 No □ 2	El padre □<br>Si □ 1 No □ 2	La madre □<br>Si □ 1 No □ 2	Otro/a (especificar): □<br>Si □ 1 No □ 2

3. **En caso afirmativo, ¿los retomará/n en un futuro próximo?**

1	Sí
2	Quizá
3	No

Sección B

1. **¿Cuánta importancia tiene para la calidad de vida de su familia que sus miembros ejerzan o se preparen para las carreras profesionales que desean?**

5	Mucha importancia
4	Bastante importancia
3	Algo de importancia
2	Poca importancia
1	Casi sin importancia

2. **¿Hay oportunidades para que los miembros de su familia ejerzan o se formen en las carreras que desean y asistan a los centros que quieren?**

5	Muchas
4	Bastantes
3	Algunas
2	Muy pocas
1	Ninguna

3. **¿Se esfuerzan los miembros de su familia por desarrollar su formación y/o carreras profesionales?**

5	Mucho
4	Bastante

3 Algo
2 Muy poco
1 Nada

4. **¿En qué medida los miembros de su familia han podido prepararse y formarse en las carreras que querían?**

5 Mucho
4 Bastante
3 Algo
2 Muy poco
1 Nada

5. **En un futuro próximo, es probable que la capacidad de su familia para ejercer y prepararse para las carreras profesionales que quieren…**

5 Mejore mucho
4 Mejore
3 Sea similar
2 Empeore
1 Empeore mucho

6. **¿En qué medida su familia está satisfecha con las carreras profesionales de los miembros de su familia y su capacidad para prepararse en esas carreras?**

5 Muy satisfecha
4 Satisfecha
3 Ni satisfecha ni insatisfecha
2 Insatisfecha
1 Muy insatisfecha

8. Ocio y tiempo libre

En esta sección, tenga en cuenta las actividades de ocio y tiempo libre de su familia en su conjunto.

Sección A

1. **¿Con qué frecuencia los miembros de la familia realizan de forma conjunta actividades de ocio y tiempo libre?**

5 Siempre
4 Casi siempre
3 Frecuentemente
2 Ocasionalmente
1 Nunca

2. ¿Con qué frecuencia los miembros de la familia realizan de forma individual actividades de ocio y tiempo libre?

- 5 Siempre
- 4 Casi siempre
- 3 Frecuentemente
- 2 Ocasionalmente
- 1 Nunca

3. ¿Con qué frecuencia participa el familiar/es con enfermedad neurodegenerativa en las actividades de ocio y tiempo libre de su familia?

- 5 Siempre
- 4 Casi siempre
- 3 Frecuentemente
- 2 Ocasionalmente
- 1 Nunca

Sección B

1. ¿Cuánta importancia tienen el ocio y el tiempo libre para la calidad de vida de su familia?

- 5 Mucha importancia
- 4 Bastante importancia
- 3 Algo de importancia
- 2 Poca importancia
- 1 Casi sin importancia

2. ¿Hay oportunidades para que los miembros de su familia participen en actividades de ocio y tiempo libre?

- 5 Muchas
- 4 Bastantes
- 3 Algunas
- 2 Muy pocas
- 1 Ninguna

3. ¿Se esfuerzan los miembros de su familia para participar en actividades de ocio y tiempo libre?

- 5 Mucho
- 4 Bastante
- 3 Algo
- 2 Muy poco
- 1 Nada

4. **¿En qué medida los miembros de su familia participan en actividades de ocio y tiempo libre?**

- 5 Mucho
- 4 Bastante
- 3 Algo
- 2 Muy poco
- 1 Nada

5. **En un futuro próximo, es probable que el ocio y tiempo libre de su familia…**

- 5 Mejore mucho
- 4 Mejore
- 3 Sea similar
- 2 Empeore
- 1 Empeore mucho

6. **¿En qué medida su familia está satisfecha con el ocio y tiempo libre?**

- 5 Muy satisfecha
- 4 Satisfecha
- 3 Ni satisfecha ni insatisfecha
- 2 Insatisfecha
- 1 Muy insatisfecha

9. Interacción en la comunidad

Por comunidad entendemos el sentido de pertenencia a un grupo de personas y/o lugares. En esta sección tenga en cuenta la interacción comunitaria de su familia en su conjunto.

Sección A

1. **¿Su familia ha padecido alguna forma de discriminación en su entorno por el hecho de tener un familiar con enfermedad neurodegenerativa?**

- 1 Sí
- 2 No

2. **¿En qué tipo de población reside su familia?**

- 7 Menor o igual 2.000 habitantes
- 6 Entre 2.001 y10.000 habitantes
- 5 Entre 10.001 y 50.000 habitantes
- 4 Entre 50.001 y 100.000 habitantes
- 3 Entre 100.001 y 400.000 habitantes
- 2 400.001 a 1.000.000 habitantes
- 1 Más de 1.000.000 habitantes

3. ¿Le gusta vivir en su población?

- 5 Mucho
- 4 Bastante
- 3 Algo
- 2 Muy poco
- 1 Nada

4. ¿La enfermedad neurodegenerativa ha sido motivo de traslado a una población mayor?

- 1 Sí
- 2 No

5. ¿En el futuro piensan hacerlo?

- 1 Sí
- 2 No

Sección B

1. ¿Cuánta importancia tiene para la calidad de vida de su familia la interacción de sus miembros con personas y lugares de su comunidad?

- 5 Mucha importancia
- 4 Bastante importancia
- 3 Algo de importancia
- 2 Poca importancia
- 1 Casi sin importancia

2. ¿Hay oportunidades para que los miembros de su familia interactúen con personas y lugares de su comunidad?

- 5 Muchas
- 4 Bastantes
- 3 Algunas
- 2 Muy pocas
- 1 Ninguna

3. ¿Se esfuerzan los miembros de su familia para interactuar con personas y lugares de su comunidad?

- 5 Mucho
- 4 Bastante
- 3 Algo
- 2 Muy poco
- 1 Nada

4. **¿En qué medida interactúa su familia con personas y lugares de su comunidad?**

5	Mucho
4	Bastante
3	Algo
2	Muy poco
1	Nada

5. **En un futuro próximo, es probable que la interacción de su familia con las personas y lugares de su entorno…**

5	Mejore mucho
4	Mejore
3	Sea similar
2	Empeore
1	Empeore mucho

6. **¿En qué medida su familia está satisfecha de la interacción con las personas y lugares de su entorno?**

5	Muy satisfecha
4	Satisfecha
3	Ni satisfecha ni insatisfecha
2	Insatisfecha
1	Muy insatisfecha

10. Calidad de vida familiar general

1. **En general, ¿cómo considera la calidad de vida de su familia?**

5	Excelente
4	Buena
3	Aceptable
2	Mala
1	Muy mala

2. **En general, ¿en qué medida está usted satisfecho con la calidad de vida de su familia?**

5	Muy satisfecho
4	Satisfecho
3	Ni satisfecho ni insatisfecho
2	Insatisfecho
1	Muy insatisfecho
